# Spontaneous Bilateral Drusen Regression Without Atrophy in an Octogenarian With Gilbert’s Syndrome: A Case Report

**DOI:** 10.7759/cureus.80993

**Published:** 2025-03-22

**Authors:** Kurt Schimmelbusch, Micaela Koci, Dean Hainsworth, David T Brockbank

**Affiliations:** 1 Ophthalmology, Revere Health, Provo, USA; 2 Ophthalmology, University of Missouri, Columbia, USA

**Keywords:** antioxidant, bilirubin, chromophore, drusen, dry macular degeneration, gilbert’s syndrome, hyperbilirubinemia, optical coherence tomography, phycocyanobilin, spirulina

## Abstract

We document significant drusen regression without atrophy (DRwoA) in a patient with dry age-related macular degeneration (AMD) and concomitant hyperbilirubinemia. Serial optical coherence tomography (OCT) and fundus images were used to monitor drusen progression in the patient. Fundus photography of the macula revealed a dramatic improvement in visible drusen in each eye over the course of 2020-2023. Only a few small drusen were visible in the right eye, while moderate to intermediate-to-large soft drusen were present in the left eye. OCT imaging also demonstrated significant improvement in drusen in both eyes compared to previous years. Average foveal thickness measurements from 2018 to 2023 showed a significant and steady decline, from 298 µm to 291 µm in the right eye and from 305 µm to 273 µm in the left eye. We report significant drusen regression in a patient with Gilbert’s syndrome and dry AMD. Further investigation into the prevalence and severity of AMD in patients with Gilbert’s syndrome is warranted. Additionally, further study of the potential therapeutic application of Spirulina in treating inflammatory conditions such as AMD is reasonable.

## Introduction

Dry age-related macular degeneration (AMD) is characterized by the progressive deterioration of the retinal pigment epithelial (RPE) cells of the macula, with sub-RPE accumulation of drusen, resulting in loss of RPE tight junctions and apoptosis of photoreceptors, apparent on histology [1]. This deterioration has been attributed to oxidative stress and aberrant activation of the complement cascade. Antioxidant supplements have been proposed to minimize oxidative damage in AMD patients and slow vision loss.

Recommendations for supplementation are based on the Age-Related Eye Disease Studies (AREDS) sponsored by the National Eye Institute. Supplements contain vitamins C and E, copper, zinc, and organic pigments such as lutein and zeaxanthin (AREDS 2). Various other organic pigments have been studied, including chromophore phycocyanobilin (PhyCB), which has demonstrated protection against blue-light-induced retinal damage in mice [2]. PhyCB is a biliverdin derivative found in cyanobacteria such as Spirulina. It is similar in structure to the molecule bilirubin, which has demonstrated antioxidant activity in vivo [3].

Individuals with Gilbert’s syndrome often display elevated serum levels of unconjugated bilirubin because of an autosomal recessive mutation in the UGT1A1 gene. This gene encodes the rate-limiting enzyme responsible for the conjugation of bilirubin, which is necessary for its elimination from the body. Patients with Gilbert’s syndrome are less likely to have a variety of inflammatory conditions, including malignancy, diabetic vascular complications, and cardiovascular diseases [4-6]. Given the powerful antioxidant activity of bilirubin, Gilbert’s syndrome may confer a selective advantage in patients with AMD.

## Case presentation

We examined an 85-year-old man with Gilbert’s syndrome and dry AMD as recently as 2023, for which he has been seen annually since 2018. He reports stable vision with no distortion, flashes, or floaters. His ocular history is significant for bilateral pseudophakia, dry eye syndrome, myopia, and regular astigmatism. The patient’s medical history is remarkable for prior neoplasm of the prostate and basal cell carcinoma, osteoporosis, gastric reflux, and mixed hyperlipidemia. He reports adherence to a standard dose of AREDS 2 for the last two years, 20 mg of simvastatin daily for the last 14 years, as well as alendronate, finasteride, omeprazole, and sildenafil. He reports a “regular” diet.

**Figure 1:**
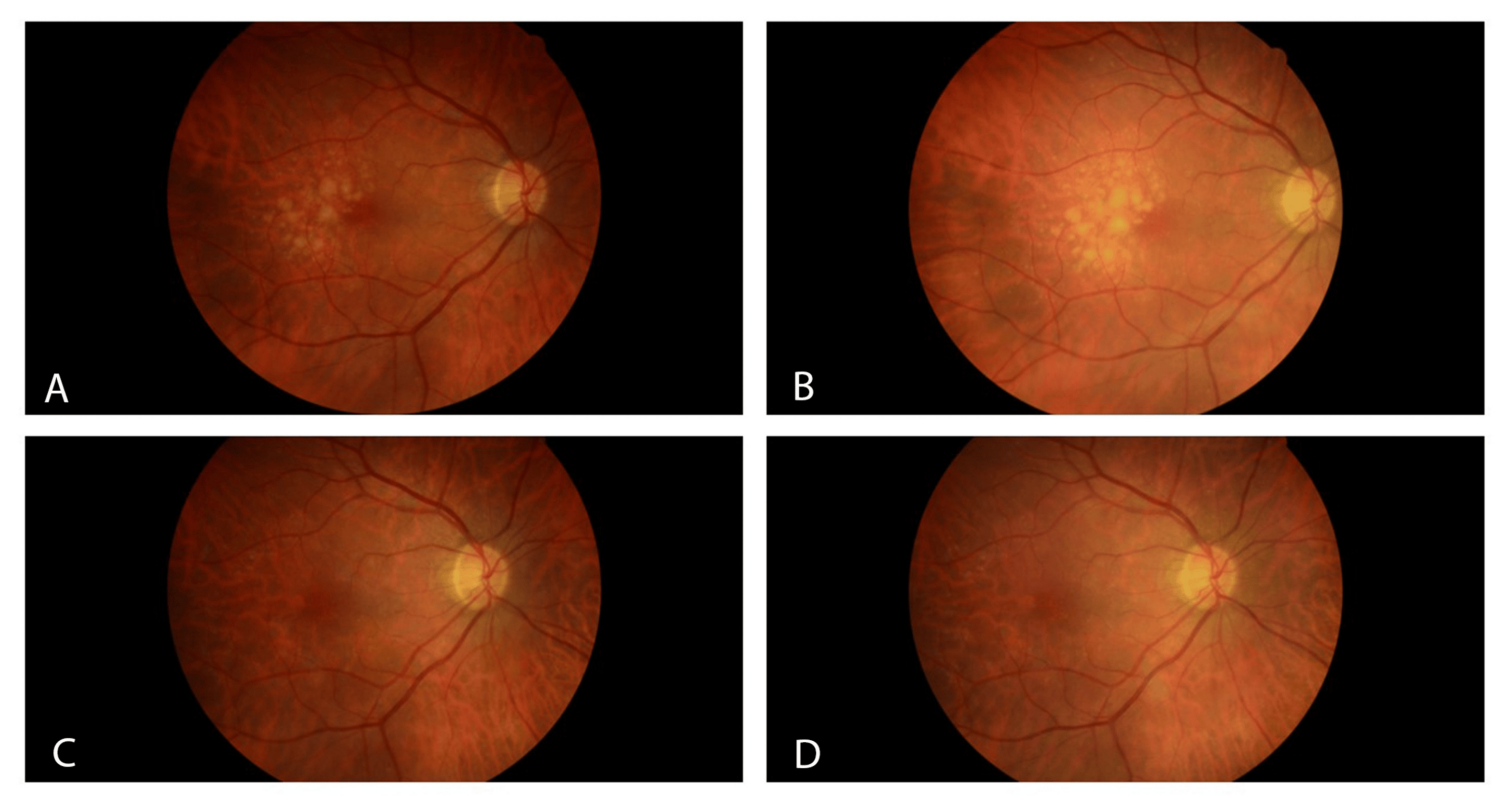
Fundus Photos of the Right Eye From 2020 to 2023 The right eye shows intermediate-stage macular drusen consistent with non-exudative macular degeneration during the years 2020 (A) and 2021 (B). There is almost a total resolution of drusen from 2021 (B) to 2022 (C). The 2023 fundus photo (D) reveals a macular appearance similar to the preceding year, with near-total resolution and only a few small drusen visible.

The best corrected visual acuity was 20/20 in the right eye and 20/25 in the left eye. Intraocular pressure was 12 mmHg in both eyes. The ocular examination was unremarkable, except for the macula, which revealed a dramatic improvement in visible drusen in each eye. There is no geographic atrophy (GA) observed. Fundus photos reveal a dramatic improvement in drusen in the right eye from 2021 to 2022 (Figure 1). 

**Figure 2:**
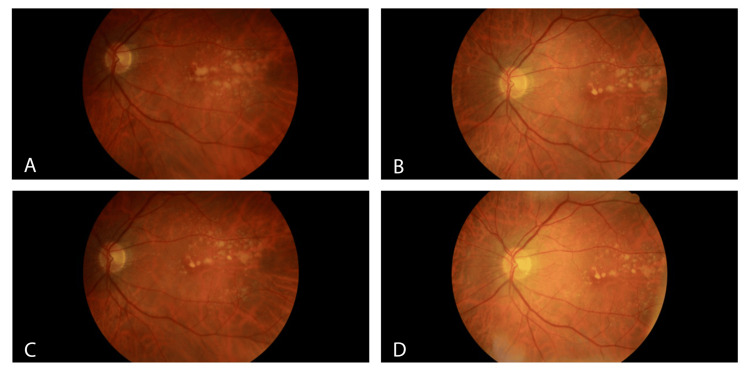
Fundus Photos of the Left Eye From 2020 to 2023 Moderate intermediate-to-large soft drusen are visible in the left eye from 2020 to 2023 (A-D, respectively). Fundus photos show a substantial reduction in drusen from 2020 (A) to 2021 (B), but interestingly, not as much from 2021 (B) to 2022 (C). The fundus photo of the left eye in 2023 (D) continues to exhibit moderate intermediate-to-large soft drusen, but still shows improvement from the previous year.

Fundus photos of the left eye show intermediate-to-large soft drusen (Figure 2). Similar to the right eye, there is a substantial reduction in visible drusen from 2020 to 2023. Photos from 2020 to 2021 show significant change, although less so from 2021 to 2022. In comparison, 2023 appears to show improvement from the previous year.

**Figure 3:**
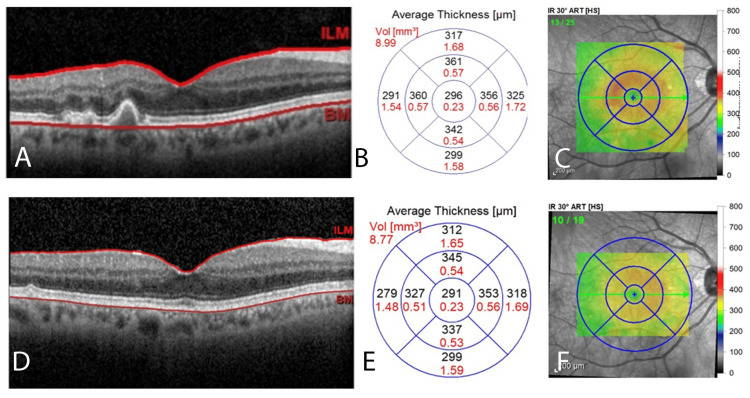
OCT Imaging With Foveolar Map of the Right Eye From 2018 to 2023 The OCT for 2018 captures a cross-section of the macula (A). OCT shows discrete, hyperreflective undulations in the RPE that are consistent with drusen deposition. Average macular thickness is depicted topographically (B) and shown in a false-color map (C). The same scheme is used to depict the macula in 2023 (D-F). Note the smooth contour of the retinal layers (D). The 2023 macular thickness map (E) shows a decrease in thickness in all regions of the rings except for the inferior perifoveolar region (unchanged). The 2023 false-color map (F) shows the reduced intensity of warmer colors in the fovea compared to 2018 (C), reflecting decreased thickness. OCT, optical coherence tomography; RPE, retinal pigment epithelium

Available optical coherence tomography (OCT) imaging from 2018 to 2019 and 2022 to 2023 shows substantial drusen reduction in both eyes. The retinal layers appear smooth and well-defined without focal areas of hyperreflectivity, drusen collapse, or RPE defects. Corresponding foveolar thickness maps show decreasing thickness. For a simple comparison, we have only included the OCT images and foveolar thickness maps from 2018 and 2023 (Figure 3). The trend observed in the interval years is consistent with that depicted in Figure 3. Baseline foveolar thickness showed a downward trend across all years, decreasing from 298 µm in 2018 to 296 µm in 2019, and further to 291 µm in both 2022 and 2023.

**Figure 4:**
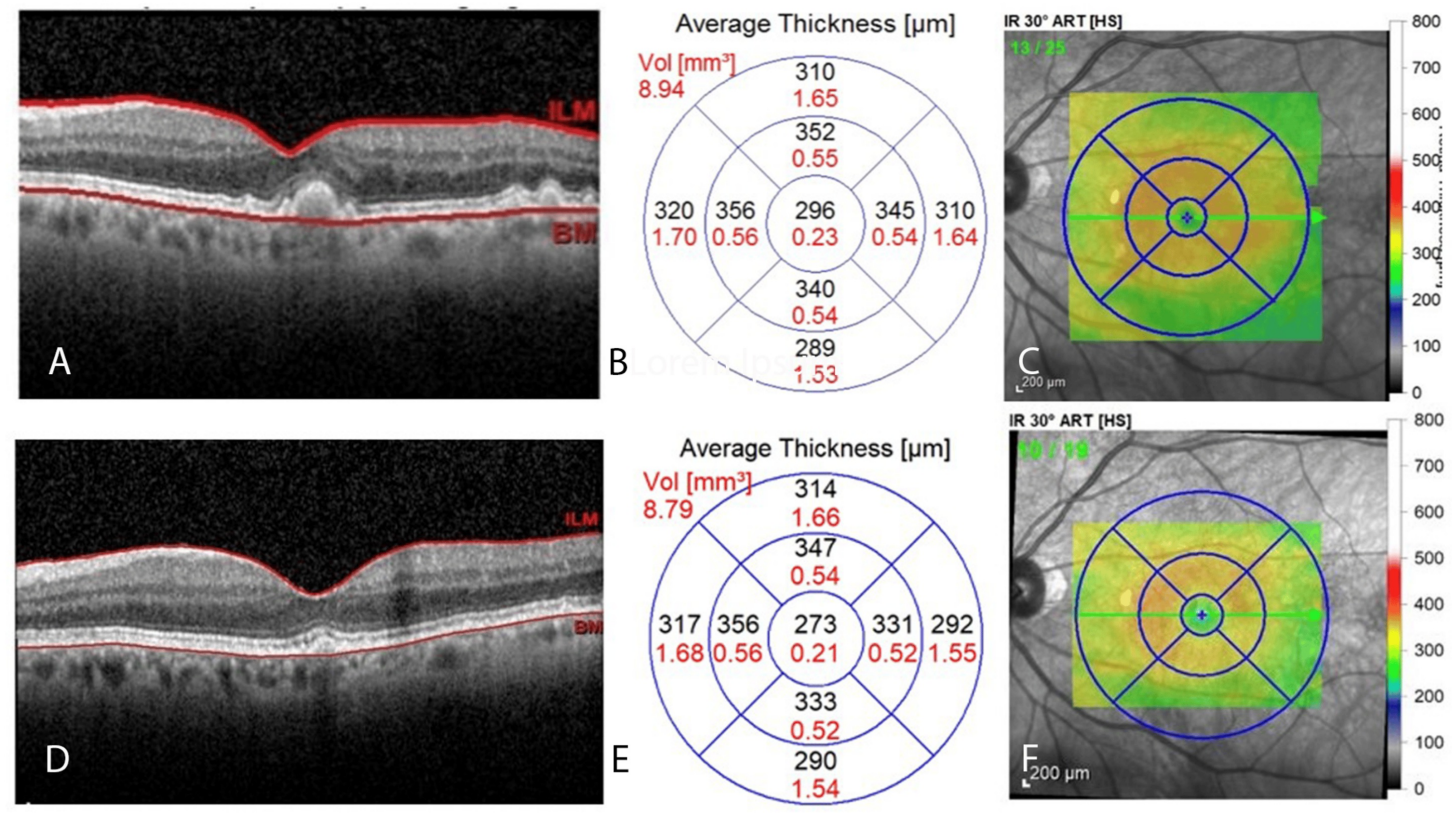
OCT Imaging With Foveolar Map of the Left Eye From 2018 to 2023 Similar to the right eye, 2018 OCT imaging of the left eye (A) reveals RPE morphology characteristic of intermediate-stage macular drusen. The corresponding quantitative (B) and qualitative (C) foveolar thickness maps for 2023 are depicted. The retinal layer (D) has fewer drusen compared to previous years. Note the dramatic decrease in average foveolar thickness in nearly all regions (E), as well as the qualitative improvement in average foveolar thickness (F). OCT, optical coherence tomography; RPE, retinal pigment epithelium

The same trend is observed in the left eye. OCT imaging shows dramatic quantitative and qualitative changes in drusen and foveolar thickness from 2018 to 2023. These changes correspond to a progressive decrease in foveolar thickness, measuring 305 µm in 2018, 296 µm in 2019, 275 µm in 2022, and 273 µm in 2023. Representative images from the initial and final years of the series are shown for comparative purposes (Figure 4). Intermediate images have been omitted to maintain conciseness.

Available sequential bilirubin levels from 2009 to 2022 demonstrate elevated total levels. Levels are consistently elevated, fluctuating year to year within a range of 0.9 mg/dL to 1.8 mg/dL, which is consistent with Gilbert’s syndrome. The patient’s total bilirubin levels peaked in 2022 at 1.8 mg/dL, nearly twice the upper limit of normal for a healthy adult. Because of the antioxidant effect of bilirubin, we propose elevated bilirubin as a contributing factor in the resolution of drusen in this patient with AMD.

## Discussion

This case demonstrates spontaneous bilateral drusen regression without atrophy (DRwoA), a phenomenon rarely reported. Interestingly, although both eyes show significant improvement, the left eye does not completely resolve like the right eye. This difference may be explained by the fact that the drusen in the left eye were larger than in the right eye prior to regression; therefore, the regression in each eye may have occurred at a similar rate. It is also possible that DRwoA regression occurred at different rates, or that the underlying mechanism(s) of regression is insufficient to resolve more advanced disease. Regardless, the patient shows no clinical signs of visual deterioration and maintains stable acuity. Imaging correlates with these clinical findings and demonstrates no visible GA on fundus photography. There are also no signs of RPE collapse on OCT, such as the presence of hyperreflective dots that precede GA in most patients with DRwoA [7]. This unique presentation in the setting of markedly elevated total bilirubin prompts consideration of the patient's history of Gilbert's syndrome and its possible influence on AMD progression.

A growing body of evidence shows that unconjugated bilirubin is a potent endogenous antioxidant that may play an important physiological role in various inflammatory diseases, including cardiovascular disease, diabetic retinopathy, and malignancy [4-6]. Although unconjugated bilirubin is not synthetically available as a potential intervention, Spirulina - a rich source of the bilirubin homolog PhyCB - has been studied in mouse models for its capacity to minimize light-induced photoreceptor damage. PhyCB demonstrated protection against light-induced photoreceptor apoptosis, subsequent visual impairment, histological changes, and reactive oxygen species (ROS) accumulation compared to the control group [8]. Spirulina, which contains the powerful bilirubin homolog PhyCB, is a promising potential therapeutic intervention for AMD. Spirulina is available as a dietary supplement but is currently only approved by the Food and Drug Administration (FDA) for use as a color additive. Research investigating the role of antioxidants in AMD suggests that their protective effects may involve the inhibition of nicotinamide adenine dinucleotide phosphate (NADPH) oxidase [9]. Viral delivery of small interfering ribonucleic acid (RNA) to p22phox, a critical membrane component of NADPH oxidase, has been shown to inhibit choroidal neovascularization in a mouse model of AMD, confirming the role of NADPH oxidase in ROS production and its potential as a therapeutic target [10].

Our data show elevated bilirubin levels dating back to 2009. However, it is unclear what the patient's exact bilirubin levels were at the time of his AMD onset. Although Gilbert's syndrome involves fluctuating bilirubin levels, the patient’s bilirubin levels have likely been chronically elevated throughout his lifetime. We acknowledge that this challenges the role of unconjugated bilirubin in the patient’s presentation, as he developed dry AMD de novo. Still, many degenerative diseases, such as multiple sclerosis (MS), are known to occur despite certain selective genetic advantages. For example, individuals with certain protective haplotypes (HLA-DRB1) may still develop MS, albeit with a milder, relapsing form of demyelinating disease [11]. Another example is retinitis pigmentosa (RP), the severity of which varies depending on genotype [12]. Like MS and RP, AMD is multifactorial. Thus, it is likely that both environmental and internal factors contribute to the complex pathogenesis of AMD. We speculate that fluctuating antioxidant levels may mitigate oxidative damage and stabilize or reduce drusen size over time.

Additional factors to consider in the patient’s presentation include his use of statins and AREDS supplements, as well as the dynamic natural history of drusen. Regarding the former, although both statins and AREDS supplements possess antioxidant activity, neither has been definitively shown to decrease drusen presence in AMD, and recent meta-analyses remain inconclusive regarding the magnitude of their protective effect [13]. Regarding the natural history of drusen, they have been observed to decrease in size over time. However, regression is generally associated with RPE atrophy and tends to occur at an advanced stage of the disease process [14]. The dramatic DRwoA in the absence of RPE collapse suggests more than the occasional natural regression observed over many years in some patients with advanced disease. Given the powerful antioxidant capacity of bilirubin and the favorable association of Gilbert's syndrome with other diseases, we hypothesize that the patient's elevated bilirubin is a contributing factor in the patient's resolution.

## Conclusions

An extensive literature review supports the powerful antioxidant capacity of unconjugated bilirubin and related analogs in decreasing the risk of developing a variety of inflammatory conditions. While the clinical and prognostic implication of drusen disappearance remains unclear, the patient’s visual acuities have remained stable over many years. These findings suggest the potential value of targeting drusen reduction as an endpoint for therapeutic intervention. Further investigations of the prevalence and severity of AMD in Gilbert’s syndrome patients are reasonable. Additionally, further study of the potential therapeutic application of Spirulina in treating inflammatory conditions, such as AMD, may prove insightful.
